# Vehicle Localization Using 3D Building Models and Point Cloud Matching

**DOI:** 10.3390/s21165356

**Published:** 2021-08-09

**Authors:** Augusto Luis Ballardini, Simone Fontana, Daniele Cattaneo, Matteo Matteucci, Domenico Giorgio Sorrenti

**Affiliations:** 1 Dipartimento di Informatica, Sistemistica e Comunicazione (DISCO), Università degli Studi di Milano-Bicocca, 20126 Milan, Italy; augusto.ballardini@uah.es (A.L.B.); simone.fontana@unimib.it (S.F.); domenico.sorrenti@unimib.it (D.G.S.); 2 Computer Engineering Department, Universidad de Alcalá, 28805 Alcala de Henares, Spain; 3 Computer Science Department, Albert-Ludwigs-Universität Freiburg, 79110 Freiburg im Breisgau, Germany; cattaneo@informatik.uni-freiburg.de; 4 Dipartimento di Elettronica Informazione e Bioingegneria (DEIB), Politecnico di Milano, 20133 Milan, Italy

**Keywords:** urban vehicle localization, point cloud processing, autonomous vehicle, robot perception

## Abstract

Detecting buildings in the surroundings of an urban vehicle and matching them to building models available on map services is an emerging trend in robotics localization for urban vehicles. In this paper, we present a novel technique, which improves a previous work by detecting building façade, their positions, and finding the correspondences with their 3D models, available in OpenStreetMap. The proposed technique uses segmented point clouds produced using stereo images, processed by a convolutional neural network. The point clouds of the façades are then matched against a reference point cloud, produced extruding the buildings’ outlines, which are available on OpenStreetMap (OSM). In order to produce a lane-level localization of the vehicle, the resulting information is then fed into our probabilistic framework, called Road Layout Estimation (RLE). We prove the effectiveness of this proposal, testing it on sequences from the well-known KITTI dataset and comparing the results concerning a basic RLE version without the proposed pipeline.

## 1. Introduction

Autonomous vehicles require accurate localization to navigate a road safely. The accuracy of a localization estimate obtained with Global navigation satellite system(GNSSs) is usually degraded in urban canyons, where buildings may introduce Non-line-of-sight(NLOS) or multi-path errors [1].

A comprehensive understanding of the scene around the vehicle can be exploited to improve a localization estimate and ensure safe urban navigation. Many approaches use different kinds of detected scene elements, such as buildings, lanes, or crossings areas, matching them to the same scene elements as recovered from online mapping services. Even though different techniques have been investigated to improve localization algorithms [2,3,4], the results are still far from the level required by ITS applications; thereby, localization remains a remarkable challenge, as proved by many recent works [5,6,7,8], suggesting the necessity of further improvements.

Although the research community has so far been focused mostly on approaches that leverage road features, such as road markings and curbs [9,10,11], an exiting opportunity in urban contexts is represented by the exploitation of the buildings in the surrounding of the vehicle.

**Figure 1:**
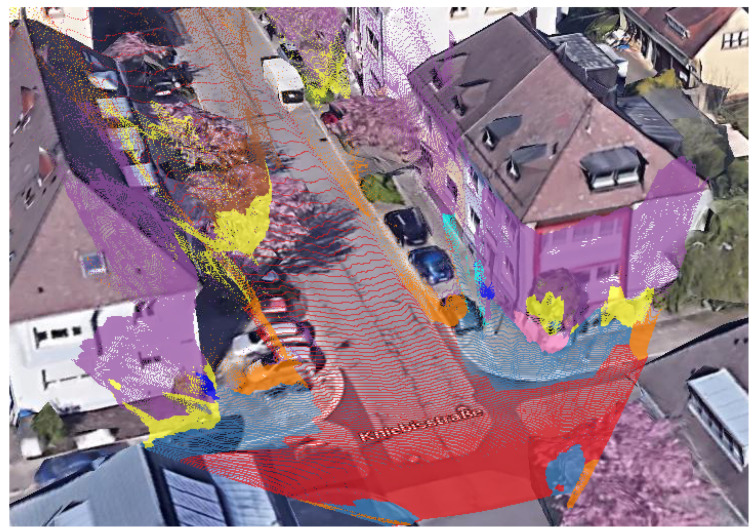
A map with the 3D buildings overlaid with the Point Clouds generated with our pipeline.

This paper presents an ego-vehicle localization method specifically designed for urban scenarios, which exploits the 3D detection of buildings’ façades. It relies on the pixel-level stereo image segmentation and 3D reconstruction capabilities of state-of-the-art pre-trained Convolutional neural network (CNNs) [12], which significantly improve the matching performances with respect to the pipeline presented in our previous work [13]. The façades, represented in terms of Point Cloud(PCs) as visible in Figure 1, can, therefore, be matched against the surrounding buildings’ outlines provided by OSM, without any preprocessing procedure or tailored scoring scheme.

With these improvements, we can now exploit, to a more significant extent, the geometry of the façades, using in our favor the primary source of NLOS or multi-path issues, i.e., the buildings themselves.

The work here presented introduces the following original contents that improve our previous work [13]:

- A revised façades detection pipeline that uses pre-trained Deep neural network(DNNs) for 3D reconstruction and pixel-level semantic segmentation;
- The 3D structure of the perceived buildings is used by the matching process, differently from our previous work, which approximated the façades by fitting infinite planes;
- A new particle scoring method that uses the GICP registration technique [14] to estimate the likelihood of the observation, i.e., of the perceived buildings, with respect to the OSM data.

**Figure 2:**
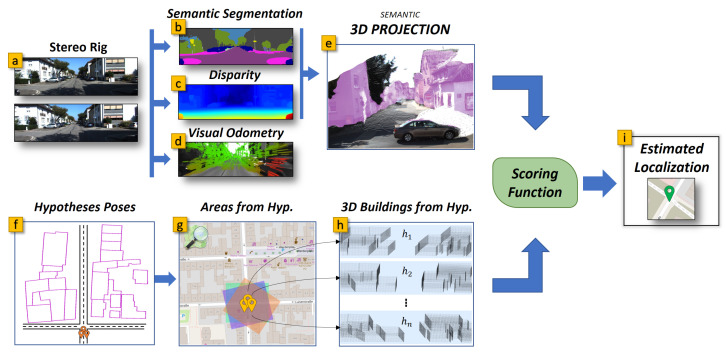
The pipeline of our proposal. A stereo rig (**a**) is used to produce a semantic segmentation (**b**), the disparity (**c**), and the visual odometry (**d**). The disparity and the semantic segmentation are used to produce a point cloud with semantic annotations (**e**). In the meanwhile, for each vehicle pose hypothesis (particle) we define a surrounding area and take the included building outlines (**f**,**g**). The outlines are then extruded in 3D (**h**) and compared against the semantic point cloud (**e**) to produce a scoring for a particle and, at the end, a localization estimate (**i**).

The proposed pipeline is depicted in Figure 2. We tested our system on five sequences from the KITTI dataset [15], evaluating the localization results using the available RTK-based Ground truth(GT).

**Figure 3:**
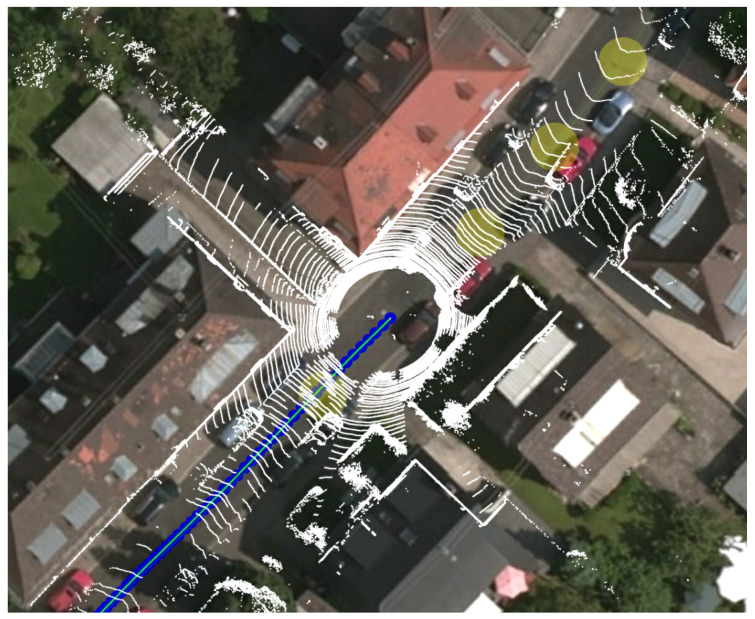
The figure depicts how we compared the velodyne data to the airbone images and the OpenStreetMap data. White points correspond to the Light detection and ranging (LIDAR) measures associated with the KITTI ground truth positions.

As the outlines of the buildings in OpenStreetMap are defined by various users and, therefore, no degree of precision is granted, we manually verified the Karlsruhe urban sequences map alignments, a subset of the sequences used in our previous work. We compared the building data with airborne images and with the overlay of the Velodyne data, using their associated GT positions. The resulting comparison showed a very accurate matching of the building outlines, see Figure 3. Consequently, we can assert that the presented technique achieves meter-level localization accuracy on sequences from the KITTI datasets that include roads with buildings in its field of view, allowing a reliable localization without the typical GNSSs degradation. This is a first step towards the use of next-generation high-definition maps, which are expected to be very precise and, therefore, will allow even greater accuracy. An overview of the existing literature on building detection and its exploitation for localization is presented in Section 2. Section 3 introduces the proposed technique while Section 4 presents the experimental activity.

## 2. Related Work

**Figure 4:**
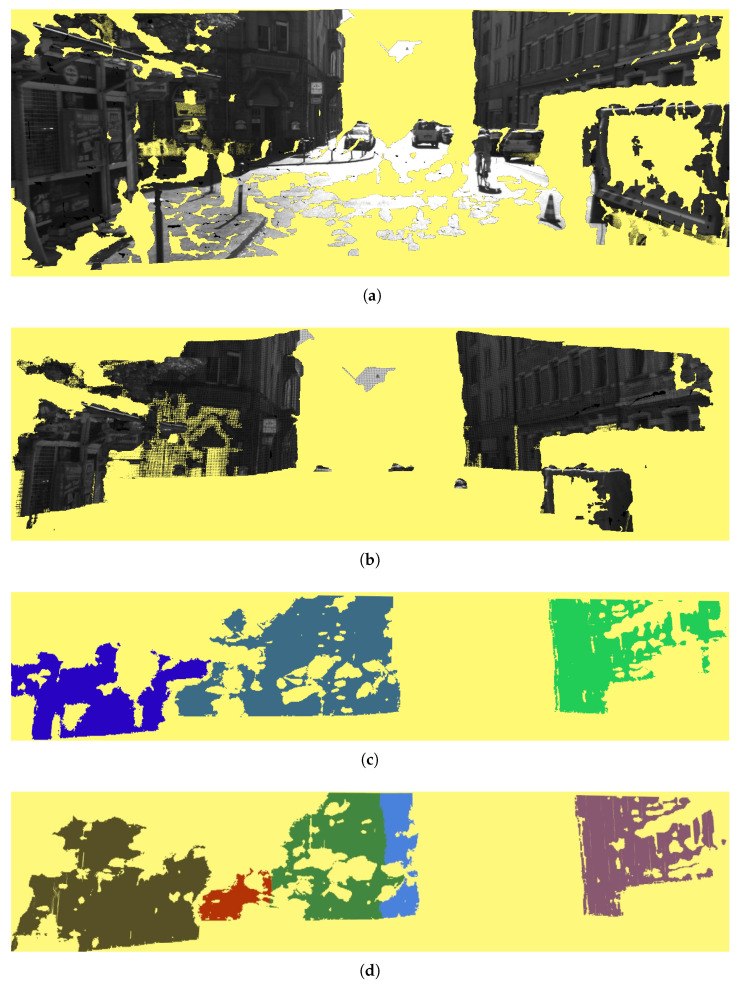
The figure depicts the steps of our previous pipeline. In yellow are the unreconstructed areas, i.e., the parts that will not be used in the reconstruction. (**a**) the point cloud from the SGMB phase, (**b**) the same image after the bounding box application. Please note that the façade structures are almost preserved. (**c**) depicts the results of the region growing segmentation algorithm, while in (**d**) the results after the final RANSAC iteration. The reconstruction quality and façade segmentation are poor in comparison with the current state-of-the-art CNN solutions shown in Figure 1.

The problem of vehicle localization has been extensively investigated in the last decades. Although commercial solutions commonly exploit GNSSs receivers in combination with Inertial navigation system(INSs) and digital maps, such as in Siemens’s navigation system [16], nowadays the research community proposes to exploit the large set of scene elements that can be found in typical driving scenarios. These scene elements could be, e.g., road markings [17], traffic lights [18], or even roundabouts and intersections [19,20,21,22]. From a technical perspective, the existing localization approaches can be divided into two main categories. On the one hand, algorithms relying on LIDARs [8,23] benefit from the great accuracy of the measures provided by these kinds of sensors, but their application is still hampered by the large sensor price. On the other hand, computer vision systems have gained importance, as the advancements in the algorithms have been impressive, and their accessibility has also increased. These systems compensate for the reliability of GNSSs either by including algorithms aimed at mitigating the NLOS problem of GPS receivers [24], by exploiting the road graph with *lock-on-road* techniques, or by roughly exploiting buildings data provided by mapping services [25,26,27,28]. In [29] the author proposes a technique to detect the 3D geometry of the buildings by fitting planes to single images of façades. Corso [5] and Delmerico et al. [30] proposed a discriminative model to answer the question: *Is this pixel part of a building façade, and if so, which façade?* They fuse pixel-level classification and surface analysis using a Markov Random Field. Even though many image-based approaches, such as the one proposed by Baats et al. [31], are closely related to the *place recognition* problem, they can still be used for vehicle localization. A comprehensive survey is available in the work of Musialski et al. [32]. In our previous work [13], we proposed a standard computer vision pipeline that took advantage of the building façades and the OpenStreetMap data to improve the localization of a vehicle driving in an urban scenario. Without using any CNN technique, our proposal leveraged images from a stereo rig mounted on a vehicle to produce a mathematical representation of the buildings’ façades within the field of view, as visible in Figure 4. This representation was matched against the outlines of the surrounding buildings as they were available on OpenStreetMap. From a technical perspective, this pipeline involved several steps, including:

- A 3D stereo reconstruction phase using the Semi-global block matching (SGBM) algorithm available in the OpenCV library [33];
- A 3D preprocessing stage aimed at refining the noisy data produced with SGBM and surface normal computation;
- A façade detection phase, involving the region growing segmentation algorithm implemented within the PCL library, followed by a clustering phase on the resulting 3D point cloud;
- A model fitting phase in which we took into consideration only regions perpendicular to the road surface;
- The extraction of the buildings’ outlines from the OpenStreetMap database;
- Finally, a step during which we compare the buildings’ outlines with the detected façades.

Since the results of each of the algorithms used in the blocks mentioned above greatly depend on a specific parameterization, an extensive experimental and tuning activity was required. Regardless of the results achieved with this system, the reliability of the underlying algorithms nowadays has been outperformed by CNN-based approaches. Indeed, the most recent works on scene understanding based on CNNs outperform most previous state-of-the-art solutions. Therefore, the work proposed in this paper leverages CNNs for pixel-level analysis. Significant performance gains have been brought in by CNNs also in the field of 3D reconstruction. Although it has been initially proposed to replace the four common stereo algorithm steps [34], according to the Stereo 2015 Benchmark [35], the latest end-to-end CNNs perform better than other kinds of approaches. Since the aim of this research is the evaluation of localization performance gains using 3D information, we opted for the best state-of-the-art reconstruction technique represented nowadays by CNNs approaches [36]. It is worth mentioning that any 3D reconstruction system would fit the purpose. For example, one far more expensive technique might be considering 3D LIDAR sensor coupled with an unavoidable preprocessing phase aimed at segmenting points corresponding to the buildings.

Matching a reconstructed point cloud to a point cloud obtained from a map service means to align the two clouds, a problem usually called *point clouds registration*. This is a well-known problem whose solutions, traditionally, are divided into two categories: either local or global registration techniques. Both categories aim at finding the rototranslation that best aligns the two point clouds. However, the first category is aimed at finding a very precise alignment, but usually requires an initial guess, that is the two clouds need to be already roughly aligned. The second category, instead, can find an alignment without any initial guess, but the results are usually much less accurate.

Algorithms in the first category are mostly derived from the Iterative closest point(ICP) algorithm [37,38], one of the first approaches to point clouds registration. The idea behind ICP is to approximate the correspondences between points, which usually are unknown, using a nearest-neighbor-based policy. However, this technique guarantees no convergence and tends to return a local optimum, instead of a global one. Many variants of ICP have been proposed, either aimed at speeding the algorithm or at improving the result [39].

A notable one is generalized ICP (GICP) [14], which incorporates the covariance of the points into the standard ICP error function and, by doing so, obtains much better results [40], although at the expense of computational time.

Another variant of ICP, developed to better deal with noise and outliers, is probabilistic Point Clouds registration (PPCR) [41]. It was derived by applying statistical inference techniques on a probabilistic model. PPCR is derived from ICP, however, to each point in the source point cloud it associates a set of points in the target point cloud (rather than one, like ICP); each association is then weighted so that the weights form a probability distribution. The result is an algorithm that is more robust than ICP.

Algorithms coming from the global registration category are often feature-based. These algorithms work by matching features among the two point clouds, similar to what is usually completed with feature matching between images. Traditional 3D features are PFH [42], the faster variant FPFH [43], and angular-invariant features [44]. Moreover, in recent years, neural network based features have been introduced, such as 3DMatch [45] and 3DSmoothNet [46]. Pointnetlk [47] and Pcrnet [48], instead, are neural network-based solutions that combine both the feature matching and the transformation estimation steps. Although global registration techniques do not need any initial guess, the results are usually inaccurate and need to be refined with a local registration technique.

Recently, global registration techniques that aim to achieve an accuracy comparable to that of local techniques have been introduced, such as fast global registration [49] and TEASER++ [50]. However, they have not yet been proven to outperform the best local algorithms.

For our localization component, we concentrated only on local registration techniques because our registration problem is inherently local: each vehicle pose hypothesis will have only a small displacement with respect to the actual pose of the vehicle, thus the corresponding point clouds should require only a fine alignment to the map.

## 3. Proposed Localization Pipeline

The idea behind the proposed technique is that if a vehicle is well localized, then what it currently perceives should be aligned with respect to the map. This is a well-known concept in the robot localization field, where a laser reading is often aligned with a previously acquired map to improve the localization estimate [51]. Therefore, we decided to use the same idea to improve a vehicle’s localization by using building façades on OpenStreetMap; unfortunately, at the moment, only bi-dimensional building outlines are available, although, in the near future, mapping services will likely include a detailed 3D representation of the façades of the buildings. Since, while driving, the vehicle can usually observe the buildings around it, we align a perceived 3D observation of these buildings with the building models available on OSM. The quality of this alignment is an indirect measure of the quality of the vehicle’s localization and is, therefore, used in a particle filter to score a particle pose.

### 3.1. Road Layout Estimation

RLE [28] is an algorithm based on a particle filter that uses a set of noisy measurements to estimate the pose of a vehicle, even in the absence of GNSS signal. From a technical perspective, the framework relies on the well-known *particle filtering* technique [52], allowing us to keep track of a whole set of localization hypotheses called Layout hypothesis(LH), which are, in turn, connected with a set of scene elements called Layout component(LC) that describe the hypothesized elements of the surrounding environment. Please note that throughout the remainder of this manuscript, the terms *particle* and LH are used alternatively. The particle filter estimates the state of a system in a non-parametric way that is without making any assumption on the probability distribution representing the state space. A general Layout hypothesisis composed of:

- The *Vehicle State*, in terms of 6DoF position, attitude, and speeds;
- The vector of LC, the scene elements associated with the hypothesis;
- The *score* of the layout, a value that combines the likelihoods of each of the LHs;
- The *motion model* to describe how the hypotheses evolve in time.

**Figure 5:**
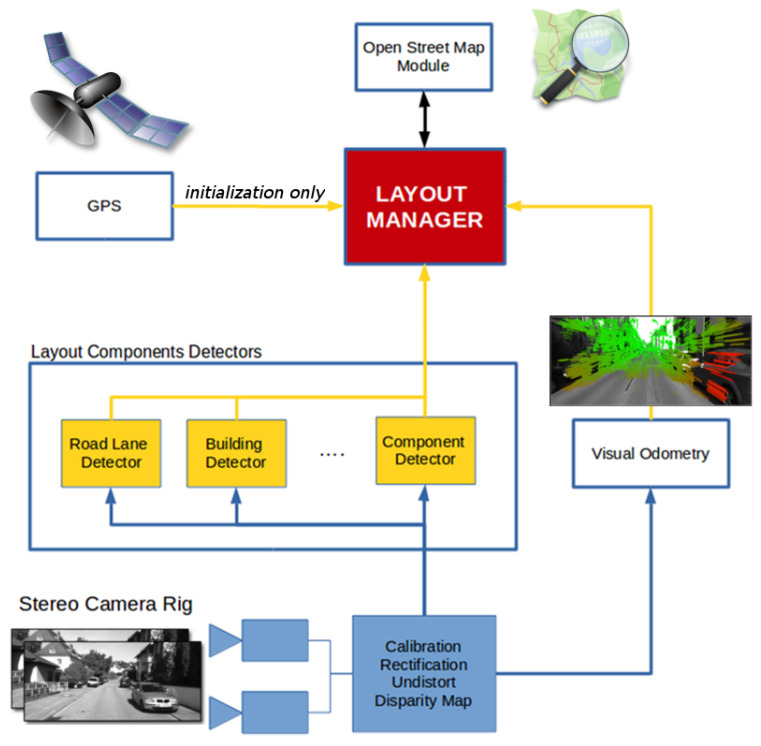
Proposed Road Layout Estimation framework schematics. The inputs are a couple of stereo images and the OpenStreetMap road network [28]. The layout manager box corresponds to the set of localization hypotheses (LHs), each of which receives inputs from ad-hoc detection pipelines. These data are used in order to create the vector of LC associated with each of the hypotheses. The contribution of this paper consists of a new building detector block, i.e., a new LC used within the LH evaluation. Intuitively, the better score the LH has, the better the localization of the vehicle is. Please notice that the GPS is used during the initialization phase only.

At each iteration of the filter, the particle’s pose and the associated components are updated according to the odometric measurements coming from the vehicle (or even from a visual odometry system) and the motion model of the LH. Then, each LH is evaluated according to the hypothesized vehicle state and the likelihood of each of the detected LCs. Conceptually, a LH should have a high score if the observation is compatible with the observer pose and the poses of the represented LCs, a low score otherwise. The LCs are the core of the scene understanding process. For road vehicles, a typical scene presents static elements such as lane markings, as well as dynamic objects such as other vehicles, see Figure 5 for an overall view of the general concept.

In this work, we propose to enrich the scene description with a new scene element for taking into account the buildings in the surrounding of the vehicle and data from OSM. From a technical perspective, we take the façades of the building as perceived by the vehicle and match them with their outlines in OSM. If the perceived buildings are well aligned with the data on OSM, then the particle represents a good estimate vehicle’s pose and, therefore, should be given a high score. Most importantly, this LC, and thus the proposed approach, should not be used alone, but it should be combined with others [20] to improve the robustness of the approach, e.g., when no building is clearly visible.

### 3.2. The Point Clouds

We decided to use Point Clouds to represent the façades of the buildings. The same type of representation has also been used for the outlines extracted from OSM, so that the two data can be aligned using standard point clouds registration techniques.

The PCs representing the façades have been produced using a CNN fed with images produced with a stereo rig. However, PCs produced with any kind of sensor could be used as no assumption is made on the characteristics of the PCs, being it the density, the presence of geometric features, or whether they are organized or not. Nevertheless, we used a stereo rig because it is a very common sensor for autonomous vehicles.

For pixel-level semantic classification, we use the dilation network proposed by Yu et al. [53], which was specifically developed for this task and has an Intersection over union(IoU) metric of 84.6 with respect to building entities.Specifically, we use the pre-trained network called *Dilation7*, which was trained on the KITTI dataset. This CNN exploits a neural network for object classification, trained on the ImageNet dataset [54], along with *de-convolutional* layers and skip-connection schemes, to improve the output with respect to the image resolution.

To fully reconstruct the scene, in addition to the semantic segmentation, we also need to reconstruct the 3D geometry. With both semantic segmentation and 3D reconstruction, we can map each classified pixel to the corresponding 3D point. According to the KITTI-STEREO-2015 benchmark [55], in the last few years CNNs demonstrated top performances also with the 3D reconstruction from stereo images task. For this reason, with respect to our previous work [13], we replaced the SGBM based approach for 3D reconstruction [33] with PSMnet [56], a recent CNN-based work. Specifically, we used the pre-trained model called *KITTI 2012*, made available by the authors. Since for these steps we used pre-trained networks, for the details and the parameters used please consult the aforementioned corresponding papers.

The core of our approach, that is, the particle scoring method, can be used in conjunction with any other technique for scene reconstruction and segmentation. We opted for the aforementioned CNNs for their performances. However, thanks to the extreme modularity of our approach, in the future these networks can be effortlessly replaced with others better performing.

**Figure 6:**
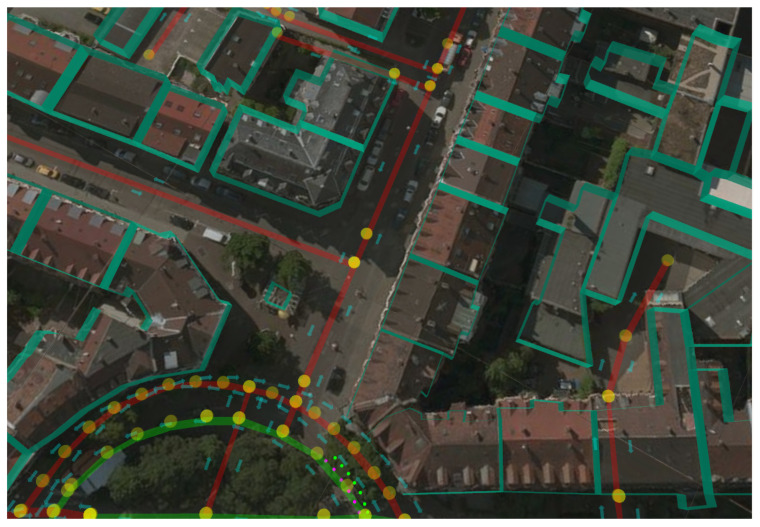
OpenStreetMap (OSM) with buildings’ outlines overlaid. The yellow dots represent the OSM *nodes*, the red lines the OSM *ways* and the light green rectangles the buildings outlines.

The second kinds of data that our technique uses are the outlines of the buildings available in OSM (Figure 6). These outlines are represented on OSM as a list of points describing the corners of a 2D representation of the façades as viewed from above. Although this information is provided by various users of the map service (thus, it is not quality checked), it is usually accurate enough to allow a fair comparison, especially in the areas of Karlsruhe. As the observed PCs are 3-dimensional, while the outlines are bi-dimensional, to align one with the other, we either have to project the 3D PCs onto a 2D plane, therefore losing some information as the façades are not just vertical planes, or to extrude the outlines in the 3D space. We opted for the second solution for a significant reason: while still not publicly available, many mapping services (such as Here or Google Maps) have already collected and are still collecting 3D representations of the buildings. This kind of representation is much more accurate than the outlines alone and carries much more detail. For example, the balconies cannot be described by a 2D representation, while the vehicle sees them and they could also provide useful anchoring points for the registration process. In the near future, when these 3D representations will be available to the public, our technique will be ready to use them and reach even higher accuracies.

To extrude the outlines, we assumed a fixed height of the buildings. This simplification usually does not bring large errors because a camera mounted on a vehicle is often not able to observe up to the top of the buildings. Although the top of buildings far from the vehicle could still be visible, these data are usually not accurate, since the errors in the stereo reconstruction increase quadratically with the distance [57]. For this reason, we used only the portion of the point cloud closer to the camera.

An important advantage of the PCs produced with the aforementioned approach is that each point is associated with a label describing what that point represents, e.g., a building, a road, or a car. In other words, we have a semantic segmentation of the PCs. For our purpose, we can therefore discard everything but the buildings, avoiding aligning other objects with the OSM data and, therefore, eliminating an important source of error of our previous approach.

### 3.3. The Registration Step

To measure the quality of a pose estimate, i.e., the score of a particle, we compare the 3D reconstruction, as perceived by the vehicle, with the buildings’ outlines from OSM (extruded in 3D). If the estimate was perfect, the two reconstructions should coincide. However, in practice, there will be a displacement between them. The farther the 3D reconstruction is from the OSM’s outlines, the worse the localization estimate is. To measure this sort of distance, we use a point clouds registration technique.

With iterative point clouds registration algorithms, the pose of a PC, called *source*, is iteratively changed to match the target cloud until some convergence criterion is met. To measure the quality of a pose estimate of the vehicle, we try to align the vehicle’s 3D scene reconstruction, used as source PC, with the outlines from OSM, used as target PC. The 3D reconstruction will then be moved by the registration algorithm to match with the OSM outlines. To measure the quality of a pose estimate, we decided to take the distance between the initial pose of the source PC and its final aligned pose. If the vehicle was perfectly localized, the source point cloud should be perfectly aligned with the target since what the vehicle perceives should be aligned with the map. On the contrary, if the registration process had to significantly move the source PC to align it with the target, it means that the pose estimate was worse than if it had to move it by a smaller distance.

Measuring the distance between two poses is a non-trivial problem since there is no shared standard method to measure the combined distance between two positions and two orientations. However, in our case, we can take advantage of the PCs associated with each iteration step of the registration process. Since we know that the *n-th* point in the source PC in the initial pose corresponds to the same *n-th* point in the final pose (roto-translating a PC preserves the order of the points), we decided to measure the distance between the two poses by using the mean distance between all the corresponding points, as described by Agamennoni et al. [41]. This is similar to what is usually performed in other research fields, when a solid is fitted on each of the poses and the mean distance between the homologous vertices of the solid in the two poses is taken as a measure of the distance between the poses [58].

Formally, given two point clouds *P* and *G*, which represent the same scene in two different poses, composed of points $p_{i}$ in *P* corresponding to points $g_{i}$ in *G*, the distance between the poses of *P* and *G* is

(1) $$\delta \left(P,G\right)=\frac{\sum_{i=0}^{n}\sqrt{∥p_{i}-g_{i}{∥}_{2}}}{n}$$

where ${∥x∥}_{2}$ is the Euclidean norm of vector x and *n* the cardinality of the point clouds.

To align the two PCs, we used generalized ICP [14] because, among the many different solutions to local point clouds registration, it provides very good results with a reasonable computational time [39,40]. Although recently new approaches to point clouds registration have been introduced, such as Teaser++ [50] and fast global registration [49], they are mainly aimed at global registration and have not been proven to outperform the best local registration algorithms, such as GICP. Nevertheless, our approach is completely agnostic with respect to the registration algorithm used: we tested it with GICP to prove its effectiveness because, in our opinion, it was the best choice when the experiments had been performed, but it can use any kind of point clouds registration algorithm. Indeed, our proposal is extremely modular: most components, such as the registration algorithm and the 3D reconstruction or the segmentation networks, can be effortlessly changed.

To increase the speed of the registration and to reduce the noise introduced during the 3D reconstruction, the PCs have been uniformly sub-sampled using a voxel grid with a leaf size of 50 cm [59]. The PCs have also been cropped to remove parts too far from the camera, that is, farther than 40 meters, and, therefore, very noisy. Since we expect the two point clouds to be very close (the pose estimates of the vehicle should not be too far from the ground truth), we allowed a maximum of 10 iterations of GICP, which proved to be a satisfactory iteration number in all cases, while keeping the computational cost very low.

The scoring function s(·) associated with the LC introduced in Section 3.1 must return a score between 0 and 1. However, the distance between two generic poses obviously does not follow this constraint. In the best case, we want the distance to be as close to 0 as possible: this corresponds to the best score for a particle since it means that it is in the right position. Therefore, a distance d=0 should correspond to the maximum score, that is d=1. The greater the distance, the lower the score should be, with very large distances corresponding to very low scores. For this reason, to evaluate a particle we used a Gaussian probability density function with zero mean and standard deviation of 100: for a particular value of distance, we take the corresponding normalized value of the density function. At the moment, there is no automatic way of choosing the standard deviation. Therefore, we performed several experiments with different values and found that the reported value of 100 led to the best results in most cases.

The Gaussian in Equation (2) formalize our informal requirements for the scoring function:

(2) $$s\left(\cdot \right)=\frac{1}{\sigma \sqrt{2\pi }}e^{-\frac{1}{2}{\left(\frac{d-\mu }{\sigma }\right)}^{2}}$$

where *d* is the distance between the two PCs as in Equation (1), μ is the mean and σ the standard deviation.

To summarize, we introduced a new layout component in the RLE framework. The associated scoring factor is the result of the alignment of a PC produced with a stereo rig and a PC obtained extruding the outlines of the surrounding buildings in OSM. The distance between the initial pose (i.e., that of the particle) of the stereo PC and the pose after the alignment is the new scoring factor: the greater the distance, the farther the particle is from the actual pose of the vehicle.

## 4. Experimental Results

We tested our approach using the KITTI dataset, which is composed of several sequences recorded with a vehicle driving in many different environments [15]. Since our approach requires buildings in the field of view of the vehicle, we used only a subset of suitable sequences and discarded the others. The sequences we used are summarized in Table 1.

For each sequence, the KITTI dataset provides a ground truth position (GT) measured with a RTK-GPS and the corresponding timestamps. Therefore, to evaluate the quality of our localization estimates, we used the Euclidean distance and the vehicle’s attitude between an estimated pose and the corresponding GT.

**Figure 7:**
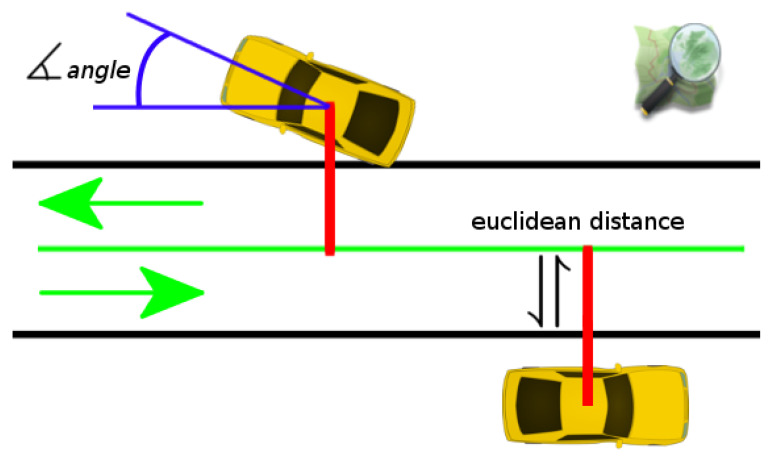
The primary *lock-on-road* component implemented in the first Road Layout Estimation framework [28]. The score of this component contributes to the LH evaluation considering the distance (the red line) with respect to the nearest road segment (the green line) and the angular misalignment (the blue arc), which also includes the road driving direction.

We compared the results of the proposed approach with those obtained using the Road Layout Estimation framework [28] with only the primary *lock-on-road* component that took into consideration both Euclidean distance and angular mismatch with respect to the actual road, see Figure 7. As described earlier, RLE is a very generic framework for vehicles localization that can exploit many kinds of components. We wanted to verify whether the component proposed in this work, which exploits buildings’ facades, effectively improves the vehicle’s localization.

Table 1 shows the mean errors between the localization estimates and the GT among each sequence. As can be seen, the proposed approach improved the mean error obtained with the primary RLE component in all but one sequence. Table 2 shows the median errors among a sequence, instead of the mean, while Table 3 shows the largest error in each sequence. The latter two tables confirm the observation made for Table 1.

Although working with the sequence *2011_09_30_drive_0028*, we noticed that there were segments where the GT positions were definitely wrong: if they were correct, the car would have been driving inside the buildings. Therefore, in Table 1 we provide results only for the first portion of that sequence, which had a realistic GT.

**Figure 8:**
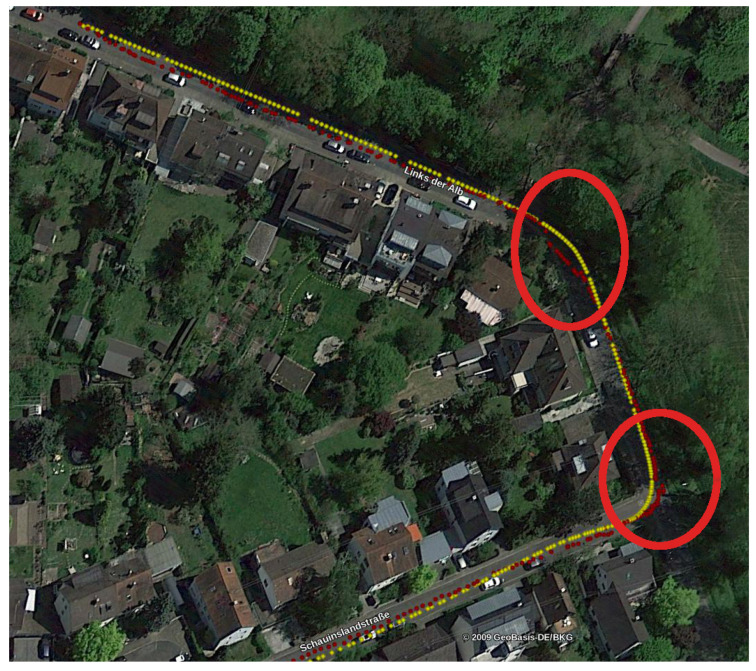
The trajectory for the *2011_09_30_drive_0028* sequence. Yellow points are the positions as measured by the GPS, red points are the position estimates of our proposal. Critical sections where surrounding buildings are not visible are circled in red.

**Figure 9:**
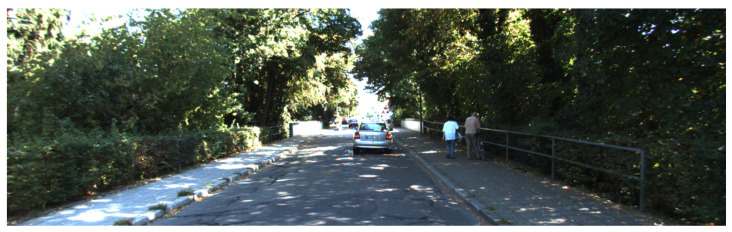
A critical section for our proposal: the vehicle is surrounded by trees and hedges.

**Figure 10:**
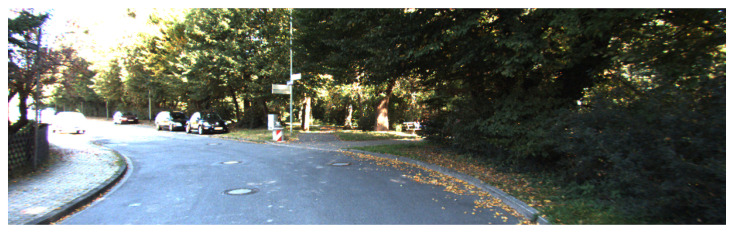
The most critical section for our proposal: the vehicle is surrounded by trees and hedges at a turn.

On sequence *2011_09_26_drive_0005* the resulting mean error is less than half the original and, very importantly, has sub-meter accuracy. Sub-meter accuracy was also attained for sequence *2011_09_26_0095*, even though the improvement with respect to the original Road Layout Estimation algorithm is less significant. On the other hand, the mean error on sequence *2011_09_30_drive_0028* is greater than that of the original approach. This is due to the fact that this sequence has large sections where no building is visible around the vehicle, either because covered by other vehicles or because there are no buildings at all. Figure 8 represents the trajectory for this sequence: yellow points are the positions as measured by the GPS, while red points are the position estimates of our proposal. As it can be seen, our pose estimate diverges from the GT mainly in areas where there are few buildings, and the vehicle is surrounded by trees and hedges. Figure 9 and Figure 10 show two frames taken from a critical section: clearly no building can be seen. RLE, without our new proposal, instead, performed well on this sequence because the vehicle drove more or less at the center of a lane during the recording of the data. This is exactly the kind of assumption that the basic RLE does, with its *lock-on-road* policy and, therefore, its better performance on this particular sequence. An essential feature of our new proposal is that, even if the pose estimate is wrong when no building can be seen, the algorithm is able to recover from these *soft* failures as soon as new useful data are received. Indeed, after the first critical section circled in red in Figure 8, the pose estimate returns to be very close to the GT.

**Figure 11:**
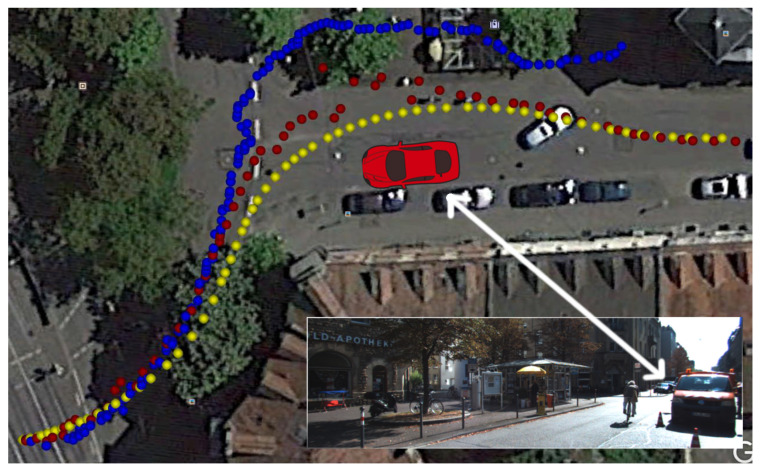
The trajectory for the *2011_09_26_drive_0005* sequence. Yellow points are the positions as measured by the GPS, red points are the position estimates of our proposal, blue points are the position estimates of RLE without the new proposal. The red car is associated with the parked truck visible in the overlaid camera frame (white arrow). As the reader will notice, the red sequence of dots matches the GPS after a short period of uncertainty while our previous work becomes lost after drifting (subsequent blue points were outside of the acceptable distance from the main road).

**Figure 12:**
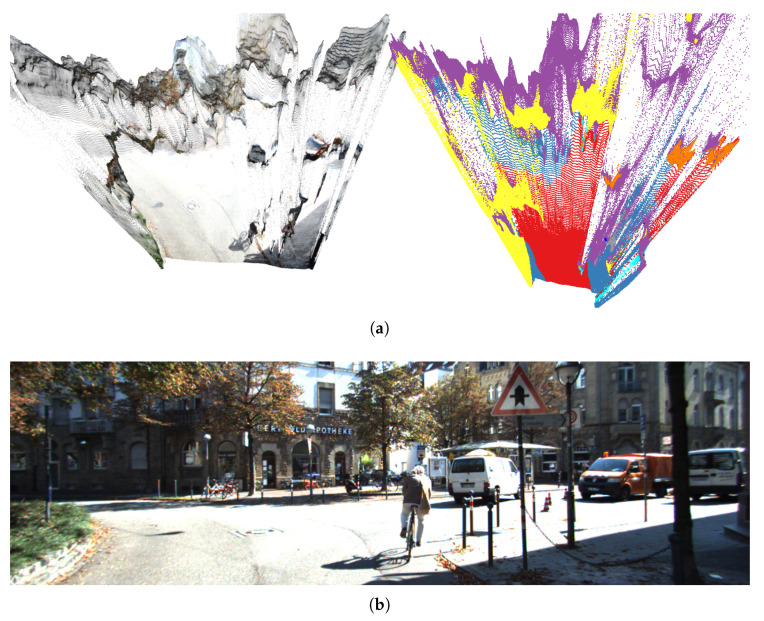
A point cloud together with the corresponding segmentation (**a**) and the corresponding RGB image (**b**) from sequence *2011_09_26_drive_0005*. The reader can appreciate that there is too much noise and distortion to obtain a proper alignment with OpenStreetMap data.

Our new proposal performed much better than the older approach when the vehicle did not strictly follow the lane. This is the case of sequence *2011_09_26_drive_0005*, whose trajectory is depicted in Figure 11. However, in this sequence too there are sections where the error with respect to the GT is not negligible. This is due to the quality of the PCs that, even though the buildings are visible, sometimes are very noisy. For example, in Figure 12a we have very noisy PCs, produced from Figure 12b, which corresponds to the area where the proposal achieved the worst results. As it can be seen, the façades of the buildings, although visible, are very noisy and distorted and, thus, cannot be properly aligned. This problem is due to the quality of the 3D reconstruction produced by the neural network we employ.

**Figure 13:**
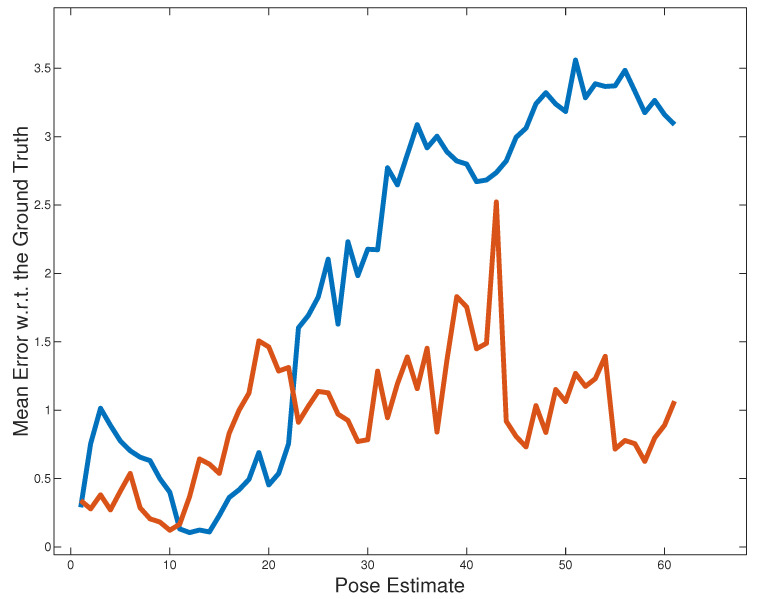
The mean error with respect to the GT for our new proposal (in red) and the old version of RLE (in blue) for the *2011_09_26_drive_0005* sequence.

However, even after such a critical situation, i.e., after crossing the roundabout and with a low-quality reconstruction of the scene, the algorithm is able to recover and correctly estimate the pose of the vehicle, achieving much better results than the previous RLE configuration, Figure 13. This is because the vehicle does not strictly follow the road lane, because of the parked truck indicated in Figure 11. In these situations, our new *building detection and registration* component adds useful information to the particle filter.

It is important to mention that, in its current development state, our proposal does not execute in real-time. To produce the results shown, we played the datasets at half their real frame rate and we used pre-computed point clouds and segmentation. For each frame, on a desktop equipped with a *GeForce GTX 1080 TI* graphics card, calculating the disparity with the neural network took 200 ms, producing a point cloud from the disparity took 20 ms per frame while producing the semantic segmentation took 500 ms per frame. It has to be noted that the latter operations, in a real autonomous vehicle, are necessary for other tasks too, such as navigation and obstacle avoidance. Therefore, these computational times are not strictly related to our approach only. Although the time spent for generating the target PC is negligible, the registration of a single pair of PCs, using a non-optimized version of GICP, took approximately 100 ms.

## 5. Conclusions

We presented a novel technique for improving autonomous vehicles’ localization in an urban scenario. Our proposal consists of a new layout component for the Road Layout Estimation framework [28] that takes into account the buildings in the surroundings of the vehicle and tries to match them with data from OpenStreetMap. The idea behind this approach is that if the vehicle is well localized, then the façades of the buildings, as seen from the vehicle in the estimated pose, should be aligned with the data in the map. For this reason, we try to align a PC obtained from state-of-the-art CNNs with that produced using the buildings’ outlines in OpenStreetMap. The more we have to move the first cloud, the worse the localization hypothesis represented by the particle is. Although we tested the approach with PCs produced using a CNN on images from the KITTI dataset, it can be used with any kind of PC. The experiments show that when buildings are visible from the vehicle’s cameras, the localization accuracy benefits from this new layout component. Moreover, as soon as 3D building data in terms of PCs or any other attributable format will be available from other mapping providers in addition to the OpenStreetMap service, our technique will be ready to exploit them. From a different point of view, it is clear that a change by the automotive industry followed by more accessible LIDAR technology should, of course, improve the effectiveness of our approach, as the actual PCs are obtained via an image-to-3D pipeline. The performances of our proposal have been measured by comparing, on sequences from the KITTI dataset, the application of the PC building detection pipeline with our previous works, which included a *lock-on-road* component only. Results show that, when buildings are visible, the proposed approach is capable of sub-meter accuracy, with remarkable robustness with respect to slight localization errors. The promising results support our belief in the RLE framework, and we envision the introduction of advanced DNNs systems to further minimize the localization error by detecting more road elements both physical, e.g., road signs, as well semantic features like intersection areas, such as in [22].

## Figures and Tables

**Table 1:** Mean results on KITTI sequences. *Original* is our previous work adapted with permission from ref. [28]. Copyright 2015 Copyright IEEE, without any *building* component, *Proposal* is this work.

Sequence	Duration [min:s]	Length [m]	Original Mean Error [m]	Proposal Mean Error [m]
2011_09_26_drive_0005	0:16	66.10	2.043	0.941
2011_09_26_drive_0046	0:13	46.38	1.269	1.168
2011_09_26_drive_0095	0:27	252.63	1.265	0.963
2011_09_30_drive_0027	1:53	693.12	2.324	1.746
2011_09_30_drive_0028	7:02	3204.46	1.353	1.716

**Table 2:** Median results on KITTI sequences. *Original* is our previous work adapted with permission from ref. [28]. Copyright 2015 Copyright IEEE, without any *building* component, *Proposal* is this work.

Sequence	Duration [min:s]	Length [m]	Original Median Error [m]	Proposal Median Error [m]
2011_09_26_drive_0005	0:16	66.10	2.307	0.925
2011_09_26_drive_0046	0:13	46.38	1.400	1.119
2011_09_26_drive_0095	0:27	252.63	1.237	0.902
2011_09_30_drive_0027	1:53	693.12	1.700	1.823
2011_09_30_drive_0028	7:02	3204.46	1.361	1.447

**Table 3:** The maximum error among any KITTI sequence. *Original* is our previous work adapted with permission from ref. [28]. Copyright 2015 Copyright IEEE, without any *building* component, *Proposal* is this work.

Sequence	Duration [min:s]	Length [m]	Original Max Error [m]	Proposal Max Error [m]
2011_09_26_drive_0005	0:16	66.10	3.560	2.522
2011_09_26_drive_0046	0:13	46.38	1.948	2.037
2011_09_26_drive_0095	0:27	252.63	3.166	2.694
2011_09_30_drive_0027	1:53	693.12	10.138	5.417
2011_09_30_drive_0028	7:02	3204.46	3.391	2.366

## Data Availability

The data presented in this study are openly available in http://www.cvlibs.net/datasets/kitti (accessed on 5 August 2021). DOI:10.1177/0278364913491297.

## Abbreviations

The following abbreviations are used in this manuscript:

RLE	Road Layout Estimation
CNN	Convolutional neural network
DNN	Deep neural network
GNSS	Global navigation satellite system
INS	Inertial navigation system
NLOS	Non-line-of-sight
LIDAR	Light detection and ranging
SGBM	Semi-global block matching
IoU	Intersection over union
ICP	Iterative closest point
OSM	OpenStreetMap
PC	Point Cloud
LC	Layout component
LH	Layout hypothesis
GT	Ground truth

## References

[1] Gu Y., Hsu L.T., Kamijo S. (2016). GNSS/Onboard Inertial Sensor Integration With the Aid of 3-D Building Map for Lane-Level Vehicle Self-Localization in Urban Canyon. IEEE Trans. Veh. Technol..

[2] Floros G., Leibe B. Joint 2D-3D temporally consistent semantic segmentation of street scenes. Proceedings of the 2012 IEEE Conference on Computer Vision and Pattern Recognition.

[3] Fernández C., Izquierdo R., Llorca D.F., Sotelo M.A. A Comparative Analysis of Decision Trees Based Classifiers for Road Detection in Urban Environments. Proceedings of the 2015 IEEE 18th International Conference on Intelligent Transportation Systems.

[4] Hummel B., Thiemann W., Lulcheva I., Cohn A.G., Hogg D.C., Möller R., Neumann B. (2008). Scene Understanding of Urban Road Intersections with Description Logic. Logic and Probability for Scene Interpretation.

[5] Corso J.J. Discriminative modeling by Boosting on Multilevel Aggregates. Proceedings of the 2008 IEEE Conference on Computer Vision and Pattern Recognition.

[6] Hentschel M., Wagner B. Autonomous robot navigation based on OpenStreetMap geodata. Proceedings of the 13th International IEEE Conference on Intelligent Transportation Systems.

[7] Larnaout D., Gay-Belllile V., Bourgeois S., Dhome M. (2014). Vision-Based Differential GPS: Improving VSLAM/GPS Fusion in Urban Environment with 3D Building Models. IEEE Int. Conf. 3D Vis. (3DV).

[8] Ruchti P., Steder B., Ruhnke M., Burgard W. Localization on OpenStreetMap data using a 3D laser scanner. Proceedings of the 2015 IEEE International Conference on Robotics and Automation (ICRA).

[9] Fernández C., Llorca D.F., Stiller C., Sotelo M.A. Curvature-based curb detection method in urban environments using stereo and laser. Proceedings of the 2015 IEEE Intelligent Vehicles Symposium (IV).

[10] Tao Z., Bonnifait P., Frémont V., Ibañez-Guzman J.I. Mapping and localization using GPS, lane markings and proprioceptive sensors. Proceedings of the 2013 IEEE/RSJ International Conference on Intelligent Robots and Systems.

[11] Schreiber M., Knöppel C., Franke U. LaneLoc: Lane marking based localization using highly accurate maps. Proceedings of the 2013 IEEE Intelligent Vehicles Symposium (IV).

[12] Goodfellow I., Bengio Y., Courville A. (2016). Deep Learning.

[13] Ballardini A.L., Cattaneo D., Fontana S., Sorrenti D.G. Leveraging the OSM building data to enhance the localization of an urban vehicle. Proceedings of the 2016 IEEE 19th International Conference on Intelligent Transportation Systems (ITSC).

[14] Segal A., Haehnel D., Thrun S. (2009). Generalized-icp. Robot. Sci. Syst..

[15] Geiger A., Lenz P., Stiller C., Urtasun R. (2013). Vision meets Robotics: The KITTI Dataset. Int. J. Robot. Res. (IJRR).

[16] Obradovic D., Lenz H., Schupfner M. (2007). Fusion of Sensor Data in Siemens Car Navigation System. IEEE Trans. Veh. Technol..

[17] Nedevschi S., Popescu V., Danescu R., Marita T., Oniga F. (2013). Accurate Ego-Vehicle Global Localization at Intersections through Alignment of Visual Data with Digital Map. IEEE Trans. Intell. Transp. Syst..

[18] Fairfield N., Urmson C. Traffic light mapping and detection. Proceedings of the 2011 IEEE International Conference on Robotics and Automation.

[19] Raaijmakers M., Bouzouraa M.E. In-vehicle Roundabout Perception Supported by a Priori Map Data. Proceedings of the 2015 IEEE 18th International Conference on Intelligent Transportation Systems.

[20] Ballardini A.L., Cattaneo D., Fontana S., Sorrenti D.G. An online probabilistic road intersection detector. Proceedings of the 2017 IEEE International Conference on Robotics and Automation (ICRA).

[21] Ballardini A.L., Cattaneo D., Sorrenti D.G. Visual Localization at Intersections with Digital Maps. Proceedings of the 2019 International Conference on Robotics and Automation (ICRA).

[22] Ballardini A.L., Hernández Á., Ángel Sotelo M. (2021). Model Guided Road Intersection Classification. arXiv.

[23] Ni K., Armstrong-Crews N., Sawyer S. Geo-registering 3D point clouds to 2D maps with scan matching and the Hough Transform. Proceedings of the 2013 IEEE International Conference on Acoustics, Speech and Signal Processing.

[24] Hsu L.T. GNSS multipath detection using a machine learning approach. Proceedings of the 2017 IEEE 20th International Conference on Intelligent Transportation Systems (ITSC).

[25] Alonso I.P., Llorca D.F., Gavilan M., Pardo S.A., Garcia-Garrido M.A., Vlacic L., Sotelo M.A. (2012). Accurate Global Localization Using Visual Odometry and Digital Maps on Urban Environments. IEEE Trans. Intell. Transp. Syst..

[26] Floros G., van der Zander B., Leibe B. OpenStreetSLAM: Global vehicle localization using OpenStreetMaps. Proceedings of the 2013 IEEE International Conference on Robotics and Automation.

[27] Xu D., Badino H., Huber D. Topometric localization on a road network. Proceedings of the 2014 IEEE/RSJ International Conference on Intelligent Robots and Systems.

[28] Ballardini A.L., Fontana S., Furlan A., Limongi D., Sorrenti D.G. A Framework for Outdoor Urban Environment Estimation. Proceedings of the 2015 IEEE 18th International Conference on Intelligent Transportation Systems.

[29] David P. (2008). Detecting Planar Surfaces in Outdoor Urban Environments.

[30] Delmerico J.A., David P., Corso J.J. Building facade detection, segmentation, and parameter estimation for mobile robot localization and guidance. Proceedings of the 2011 IEEE/RSJ International Conference on Intelligent Robots and Systems.

[31] Baatz G., Köser K., Chen D., Grzeszczuk R., Pollefeys M. (2012). Leveraging 3D City Models for Rotation Invariant Place-of-Interest Recognition. Int. J. Comp. Vis..

[32] Musialski P., Wonka P., Aliaga D.G., Wimmer M., Gool L., Purgathofer W. (2013). A Survey of Urban Reconstruction. J. Comp. Graph. Forum.

[33] Hirschmüller H. (2008). Stereo processing by semiglobal matching and mutual information. IEEE Trans. Pattern Anal. Mach. Intell..

[34] Scharstein D., Szeliski R. (2002). A taxonomy and evaluation of dense two-frame stereo correspondence algorithms. Int. J. Comp. Vis..

[35] Menze M., Heipke C., Geiger A. (2015). Joint 3D estimation of vehicles and scene flow. ISPRS Ann. Photogramm. Remote Sens. Spat. Inf. Sci..

[36] Poggi M., Kim S., Tosi F., Kim S., Aleotti F., Min D., Sohn K., Mattoccia S. (2021). On the Confidence of Stereo Matching in a Deep-Learning Era: A Quantitative Evaluation. https://arxiv.org/abs/2101.00431.

[37] Besl P.J., McKay N.D. (1992). A method for registration of 3-D shapes. IEEE Trans. Pattern Anal. Mach. Intell..

[38] Chen Y., Medioni G. (1992). Object modelling by registration of multiple range images. Image Vis. Comp..

[39] Pomerleau F., Colas F., Siegwart R., Magnenat S. (2013). Comparing ICP variants on real-world data sets. Auton. Robot..

[40] Fontana S., Cattaneo D., Ballardini A.L., Vaghi M., Sorrenti D.G. (2021). A benchmark for point clouds registration algorithms. Robot. Auton. Syst..

[41] Agamennoni G., Fontana S., Siegwart R.Y., Sorrenti D.G. Point clouds registration with probabilistic data association. Proceedings of the 2016 IEEE/RSJ International Conference on Intelligent Robots and Systems (IROS).

[42] Rusu R.B., Blodow N., Marton Z.C., Beetz M. Aligning point cloud views using persistent feature histograms. Proceedings of the 2008 IEEE/RSJ International Conference on Intelligent Robots and Systems.

[43] Rusu R.B., Blodow N., Beetz M. Fast point feature histograms (FPFH) for 3D registration. Proceedings of the 2009 IEEE International Conference on Robotics and Automation.

[44] Jiang J., Cheng J., Chen X. (2009). Registration for 3-D point cloud using angular-invariant feature. Neurocomputing.

[45] Zeng A., Song S., Nießner M., Fisher M., Xiao J., Funkhouser T. (2017). 3dmatch: Learning the matching of local 3d geometry in range scans. CVPR.

[46] Gojcic Z., Zhou C., Wegner J.D., Wieser A. The perfect match: 3D point cloud matching with smoothed densities. Proceedings of the IEEE/CVF Conference on Computer Vision and Pattern Recognition.

[47] Aoki Y., Goforth H., Srivatsan R.A., Lucey S. Pointnetlk: Robust & efficient point cloud registration using pointnet. Proceedings of the IEEE/CVF Conference on Computer Vision and Pattern Recognition.

[48] Sarode V., Li X., Goforth H., Aoki Y., Srivatsan R.A., Lucey S., Choset H. (2019). PCRNet: Point cloud registration network using PointNet encoding. arXiv.

[49] Zhou Q.Y., Park J., Koltun V. Fast global registration. European Conference on Computer Vision.

[50] Yang H., Shi J., Carlone L. (2020). Teaser: Fast and certifiable point cloud registration. IEEE Trans. Robot..

[51] Dellaert F., Fox D., Burgard W., Thrun S. Monte carlo localization for mobile robots. Proceedings of the 1999 IEEE International Conference on Robotics and Automation (Cat. No. 99CH36288C).

[52] Thrun S., Burgard W., Fox D. (2005). Probabilistic Robotics (Intelligent Robotics and Autonomous Agents).

[53] Yu F., Koltun V. (2016). Multi-Scale Context Aggregation by Dilated Convolutions. arXiv.

[54] Deng J., Dong W., Socher R., Li L.J., Li K., Fei-Fei L. Imagenet: A large-scale hierarchical image database. Proceedings of the 2009 IEEE Conference on Computer Vision and Pattern Recognition.

[55] Alhaija H.A., Mustikovela S.K., Mescheder L., Geiger A., Rother C. (2017). Augmented Reality Meets Deep Learning for Car Instance Segmentation in Urban Scenes. Brit. Mach. Vis. Conf. (BMVC).

[56] Chang J.R., Chen Y.S. (2018). Pyramid Stereo Matching Network. arXiv.

[57] Matthies L., Shafer S. (1987). Error modeling in stereo navigation. IEEE J. Robot. Autom..

[58] Mazzotti C., Sancisi N., Parenti-Castelli V., Parenti-Castelli V., Schiehlen W. (2016). A Measure of the Distance between Two Rigid-Body Poses Based on the Use of Platonic Solids. ROMANSY 21—Robot Design, Dynamics and Control.

[59] Holz D., Ichim A.E., Tombari F., Rusu R.B., Behnke S. (2015). Registration with the point cloud library: A modular framework for aligning in 3-D. IEEE Robot. Autom. Mag..

